# Direct Evidence for Cannibalistic Necrophagy as a Way of Nitrogen Recycling in Ants

**DOI:** 10.1002/ece3.72253

**Published:** 2025-10-15

**Authors:** Ádám Lőrincz, Kata Anna Bán, Tamás Maruzs, István Elek Maák

**Affiliations:** ^1^ Department of Ecology University of Szeged Szeged Hungary; ^2^ Doctoral School of Biology University of Szeged Szeged Hungary; ^3^ HUN‐REN Biological Research Centre Szeged Hungary; ^4^ Museum and Institute of Zoology, Polish Academy of Sciences Warsaw Poland

**Keywords:** corpse management, fluorescence, necrophagy, nestmate corpses, red wood ants

## Abstract

Adequate nitrogen sources are indispensable for the development and reproduction of most animals. Some observations suggest that eusocial insects, such as termites or ants, can cover the protein requirements of their growing larvae by consuming the corpses of their own nestmates, a behavior known as cannibalistic necrophagy. While termites commonly utilize this food source, its occurrence in ants remains controversial and has so far been supported only by indirect observations (e.g., substantial weight loss of corpses or the presence of gnawed‐out holes on the abdomen of the corpses). This behavior might be a crucial tool for survival under suboptimal conditions; however, long‐standing evidence supporting its presence in ants is limited. In this study, we assessed whether cannibalistic necrophagy indeed occurs in ants by offering fluorescently marked corpses to their nestmates and subsequently detecting the signal within the digestive tracts of the living ants. Our results provide direct evidence that some ant species can use corpses, a constantly available food source, to fulfill the nitrogen requirements of the colony. This food source can have a variable share in the diet of a colony, and we argue that it is mainly utilized when food availability is scarce. By enabling the recirculation of nitrogen from deceased colony members, necrophagy may contribute to the ecological and evolutionary success of ants.

## Graphical abstract

Adequate nitrogen sources are essential for the development and reproduction of most animals. In eusocial insects such as termites and ants, some observations suggest that the protein needs of growing larvae may be met by consuming the corpses of nestmates—a behavior that remains controversial in ants and is primarily supported by indirect evidence. Our results provide direct evidence that ants can indeed utilize corpses—an always‐available food source—to meet the colony's nitrogen requirements during periods of food shortage.

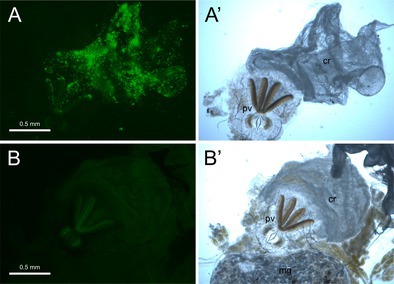

## 1 Introduction

The large colonies of eusocial insects, such as termites, bees, wasps, and ants, are heavily dependent on adequate nitrogen sources, as this element is essential for their reproduction and brood development (Jarau and Hrncir 2009). The source of nitrogen varies based on the ecological characteristics of the group in question: termites consume symbiotic fungi, as well as their dead or injured nestmates to obtain proteins (Sun and Zhou 2013; Ahmad et al. 2018); bees rely on pollen (Knox et al. 1971), while wasps and ants typically provide other arthropods as protein sources for their growing larvae (Jarau and Hrncir 2009). In temperate and boreal ecosystems, however, the populations of potential prey insects undergo drastic seasonal changes, leading to shortages of protein sources for organisms dependent upon them (Mabelis 1978; Driessen et al. 1984). During such periods, especially in early spring, some ant species engage in fierce battles with neighboring colonies and consume the fallen enemies to cover the protein requirements of their larvae (Mabelis 1978; Mori et al. 2000). When food supplies are scarce in their territory, some evidence suggests that these ants also consume the corpses of their own nestmates (Mabelis 1978; Driessen et al. 1984; Maák et al. 2020), a behavior known as cannibalistic necrophagy (Maák et al. 2020).

Necrophagy may be one way to ensure survival under suboptimal conditions (Rutkowski et al. 2019); however, the evidence supporting the existence of this behavior is limited and relies mostly on indirect observations, e.g., the presence of gnawed‐out holes on the abdomen of the corpses (Mabelis 1978; Driessen et al. 1984), or substantial weight loss of corpses (Rutkowski et al. 2019; Maák et al. 2020). Here we assessed whether cannibalistic necrophagy indeed occurs in wood ants and report direct evidence of this behavior using a fluorescence‐based technique, which involves offering fluorescently marked corpses to their fellow nestmates and subsequently detecting the signal within the digestive tracts of the living ants.

## 2 Materials and Methods

We worked with five colony fragments of a red wood ant species ( *Formica polyctena* ) that were collected from five different forest plantations near Szeged, Southern Hungary. Each colony fragment consisted of approximately 1000 workers and 200 cm^3^ of nest material. The colony fragments were housed in plastic boxes (L44 cm × W31 cm × H23 cm) with a circular opening (15 cm in diameter) on the lid of the boxes covered with a fine wired metal mesh for proper ventilation and an easier moistening and were kept under constant laboratory conditions (temperature 24°C ± 4°C; relative humidity 42%–43%; 12‐h L:D cycle). Water was always available ad libitum. One day after collection, we connected the colony fragments to a foraging arena (L60 cm × W30 cm × H15 cm) with a plastic tube (1 cm in diameter, 10 cm in length) and provided fluorescently marked food to the ants. The food consisted of 0.4 g of fluorescent dye mixed with 2 g of tuna and 4 g of honey, homogenized in 1 mL of distilled water. As a fluorescent dye, we used an ultra‐violet responsive fluorescent powder (TP40—Chartreuse, Radiant color N.V., Belgium), which is a sulphonamide–melamine–paraformaldehyde resin. We chose this dye as it has been used in similar studies and demonstrated good biocompatible properties (Okrouhlik and Foltan 2015). Ants feeding on the food source were removed; their thorax and gaster were paint‐marked on the cuticle to ensure future traceability, after which they were freeze‐killed at −20°C.

After obtaining the marked corpses (i.e., fluorescently marked in their digestive tract and paint‐marked on their cuticle), we starved the colony fragments for 2 additional days to encourage cannibalistic necrophagy. At the end of the starvation period, we separated 15 workers, including both foragers and inner‐nest workers, to ensure diversity in worker types and age groups, together with some nest material, into smaller plastic boxes (L30 cm × W16 cm × H10 cm). We then offered them five fluorescently marked nestmate corpses on a small plastic disc (10 cm in diameter). The corpses were previously washed individually in distilled water (2 mL, vortexed for 10 s, repeated 5 times) to ensure that no traces of fluorescent dye remained on their cuticle or mouthparts. Three days after being presented with fluorescently marked nestmate corpses, the living ants were anesthetized by exposure to FlyNap (triethylamine) and were dissected by opening up their gastric tergites and placing the soft tissues on a microscope slide. The crop and midgut were searched for traces of fluorescent substances under blue excitation light using a Zeiss Axio Imager.M2 fluorescent microscope with EC Plan‐Neofluar 2.5×/0.085 M27 and Fluar 5×/0.25 NA objectives. Imaging was carried out with Hamamatsu ORCA‐Flash 4.0LT (blue light illumination) and Axiocam 506 color (visible light illumination) cameras. As a control, the same procedures were applied, but the starved ants were presented with nestmate corpses previously fed on a non‐fluorescently marked food source (1:2 mixture of tuna and honey).

To ascertain that the fluorescent dye consumed by the ants remained detectable throughout our experiment, five fluorescently marked corpses (positive controls) were placed on small plastic discs (10 cm in diameter) from each colony fragment and left under similar conditions for 3 days. After this period, the corpses were dissected as described above.

## 3 Results

**FIGURE 1:**
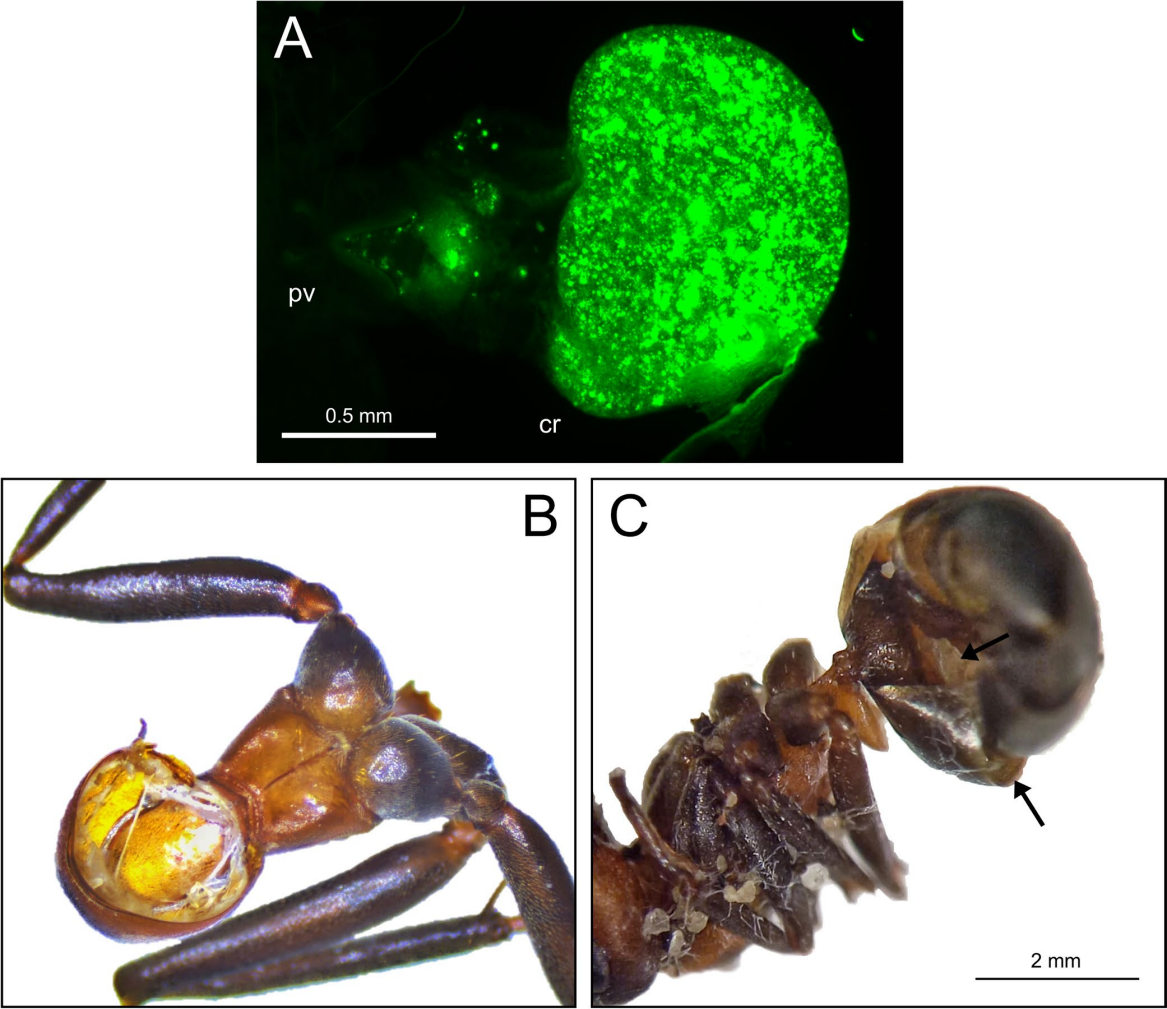
(A) Crop (cr) and proventriculus (pv) of a *Formica polyctena* worker 1 day after consuming fluorescently marked food (1:3 mixture of honey‐water). (B) Dismantled corpse of a worker that had fed on fluorescently marked food by the end of the three‐day experiment. (C) Gnawed‐out holes (indicated by black arrows) on the gaster of a worker that had fed on fluorescently marked food by the end of the experiment.

The workers directly feeding on the food source containing fluorescent dye consumed substantial amounts of fluorescent pigments and their crops showed a strong signal when dissected and illuminated by excitation light (Figure 1A). The signal persisted for the duration of the experiment and remained detectable after 3 days, although the exact location at that point could not be identified, as most organs had already decomposed.

**FIGURE 2:**
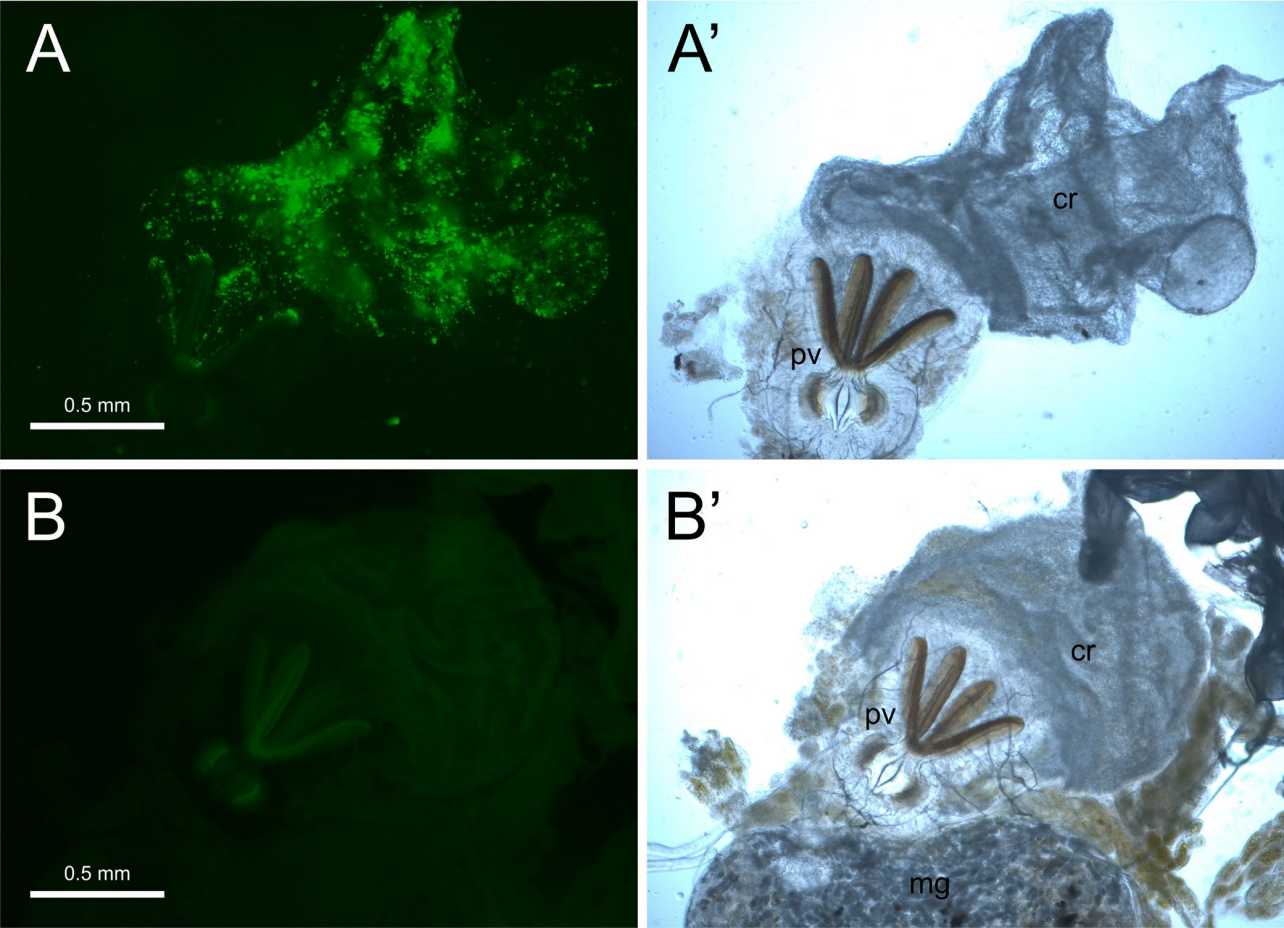
(A and A′) Crop (cr) and proventriculus (pv) of a *Formica polyctena* worker 3 days after being presented with fluorescently marked nestmate corpses. Picture A was taken using blue excitation light, whereas picture A′ was taken using visible light for illumination. (B and B′) Control experiment: Crop (cr), proventriculus (pv), and midgut (mg) of a worker 3 days after being presented with non‐marked nestmate corpses. Picture B was taken using blue excitation light, whereas picture B′ was taken using visible light for illumination.

The number of workers that survived the duration of the experiment varied substantially among colony fragments (15, 15, 14, 5, and 2, respectively). In the latter two fragments (with only five and two surviving workers), the presented nestmate corpses remained intact, and no signs of necrophagy were observed. In contrast, in the colony fragments with a much higher number of surviving workers, all offered nestmate corpses were damaged, mainly in two ways: (1) the entire corpse was dismantled, with the head, legs, and gaster chewed off, and all soft tissues removed (Figure 1B), or (2) a small hole was gnawed on the ventral side of the gaster, with the gastral sternites separated from one another (Figure 1C). The digestive tracts of some surviving workers (4, 5, and 9 workers, respectively) in these colony fragments showed a strong fluorescent signal, particularly in the crop, which in ants functions as a social stomach and serves to store food for later regurgitation to nestmates in numerous species (Hölldobler and Wilson 1990) (Figure 2A,A′). Such fluorescent signal was never observed in the crops of ants in the control group (with 15, 15, 13, 7, and 4 workers surviving the duration of the experiment), which received fluorescently non‐marked corpses as a potential food source (Figure 2B,B′).

## 4 Discussion

Our results corroborate previous findings regarding the cannibalistic behavior of red wood ants and provide direct evidence for the presence of cannibalistic necrophagy in this group, demonstrated by the transfer of fluorescently marked tissues from nestmate corpses to living workers. We found fluorescently marked individuals in three of the five colony fragments, suggesting that cannibalistic necrophagy might be a widespread behavior in this species when facing unfavorable conditions. Notably, in most cases, only a portion of the workers possessed a fluorescent signal, which might indicate inter‐individual differences in this behavior. This result, however, should be interpreted with caution, as the workers dying during the experiment were not removed to avoid disturbance of the ants. This, however, could have potentially resulted in the consumption of non‐marked corpses as well. It should also be noted that the number of workers giving a fluorescent signal does not necessarily correspond to the number of individuals directly engaging in necrophagy, as the signal may be transmitted among nestmates via oral trophallaxis. Thus, further studies are needed to ascertain whether this behavior is indeed a characteristic displayed by only a specific subset of workers within the colony and to quantify its prevalence.

Differences in worker survival in our experiment might also reflect inter‐individual differences. Although we took special care during colony fragment creation to include both inner‐nest workers and foragers, it is likely that the proportion of these worker groups, as well as the age composition, differed among fragments, which may have contributed to the observed survival differences.

On the basis of our findings, the presence of larvae is not a prerequisite for cannibalistic necrophagy, as no larvae were present in the colony fragments during our experiments. Although larval hunger is generally considered the primary factor driving the collection and consumption of protein‐rich food sources in ant colonies (Hölldobler and Wilson 1990), during periods of food deprivation, workers often also consume protein‐rich resources—mainly the brood of the colony—to keep themselves and the queens alive (a behavior known as brood cannibalism, Wilson 1971; Sorensen et al. 1983). However, while brood cannibalism is widespread among ants and other social insects (Wilson 1971; Schultner et al. 2017), cannibalistic necrophagy appears to be less common, possibly due to the high risk of infection (Maák et al. 2020).

Red wood ants are capable of surviving under unexpectedly suboptimal conditions, which is ensured by their exceptional ecological and behavioral flexibility. For example, an introduced colony has been observed to persist for more than two decades on a tiny, inhospitable island in Finland, relying on the honeydew production of aphids feeding on the only pine tree on the island (Czechowski and Vepsäläinen 2009). Another striking example illustrating the extreme resilience of this group is the survival of a colony fragment trapped inside an abandoned Soviet‐era underground nuclear bunker (Czechowski et al. 2016; Rutkowski et al. 2019; Maák et al. 2024). This curious case originates from the fact that the nest of this colony was constructed directly above a ventilation pipe, causing workers to fall into the bunker. Despite the challenges posed by the constant darkness, cold temperatures, and almost complete lack of food, many of these workers still managed to survive through the consumption of the corpses of their similarly unfortunate nestmates (Rutkowski et al. 2019). On the basis of a previous work with *F. polyctena* , the proportion of consumed corpses can reach as high as 83.9% following a period of starvation (Maák and Kiss, unpublished data). These examples demonstrate the extraordinary adaptability and perseverance of red wood ants, to which cannibalistic necrophagy contributes as a valuable tool to overcome unfavorable conditions. By allowing for the effective recirculation of nitrogen content from their fallen colony members, cannibalism might contribute to the ecological and evolutionary success of ants.

## Author Contributions

**Ádám Lőrincz:** conceptualization (equal), data curation (equal), formal analysis (equal), investigation (lead), methodology (equal), project administration (equal), visualization (equal), writing – original draft (lead), writing – review and editing (lead). **Kata Anna Bán:** formal analysis (equal), investigation (equal), methodology (equal), project administration (supporting), visualization (equal), writing – review and editing (equal). **Tamás Maruzs:** formal analysis (equal), investigation (equal), methodology (equal), visualization (equal), writing – original draft (supporting). **István Elek Maák:** conceptualization (equal), investigation (equal), methodology (equal), writing – review and editing (equal).

## Conflicts of Interest

The authors declare no conflicts of interest.

## Data Availability

All data generated during our experiments are fully included in the main text of the manuscript.
